# Selenium Status Is Associated with Inflammation in Epicardial Adipose Tissue in Elderly Patients with Coronary Artery Disease

**DOI:** 10.3390/antiox15060687

**Published:** 2026-05-29

**Authors:** Trine Baur Opstad, Fredrik Lossius Opdahl, Steen Larsen, Sissel Åkra, Sheryl Palmero, Theis Tønnessen, Svein Solheim, Jan Alexander, Urban Alehagen, Ida Gjervold Lunde

**Affiliations:** 1 Oslo Center for Clinical Heart Research, Department of Cardiology, Oslo University Hospital Ullevål, 0450 Oslo, Norway; f.l.opdahl@studmed.uio.no (F.L.O.); uxkrsi@ous-hf.no (S.Å.); sheryl.palmero@medisin.uio.no (S.P.); ssolheim@ous-hf.no (S.S.); i.g.lunde@medisin.uio.no (I.G.L.); 2 Institute of Clinical Medicine, University of Oslo, 0318 Oslo, Norway; uxthto@ous-hf.no; 3 Department of Biomedical Science, University of Copenhagen, 2200 Copenhagen, Denmark; stelar@sund.ku.dk; 4 Clinical Research Centre, Medical University of Bialystok, 15-276 Bialystok, Poland; 5 Department of Cardiothoracic Surgery, Oslo University Hospital, 0450 Oslo, Norway; 6 Norwegian Institute of Public Health, 0213 Oslo, Norway; jan.alexander@fhi.no; 7 Department of Medical and Health Sciences, Linköping University, 581 83 Linköping, Sweden; urban.alehagen@liu.se; 8 K.G. Jebsen Center for Cardiac Biomarkers, Campus Ahus, University of Oslo, 1478 Lørenskog, Norway

**Keywords:** selenium status, epicardial adipose tissue, selenoproteins, inflammation, antioxidant defense, coronary artery disease

## Abstract

Background: Inflammation in epicardial adipose tissue (EAT) contributes to cardiovascular disease through the local production of pro-inflammatory cytokines affecting the adjacent myocardium. Selenium (Se) is essential for selenoprotein-mediated antioxidant and anti-inflammatory functions. We investigated associations between Se status and inflammatory markers in EAT and in the circulation in patients with coronary artery disease (CAD). Methods: Patients with CAD undergoing coronary artery bypass grafting (*n* = 52) and valve disease patients receiving valve replacement serving as controls (*n* = 22) were included from the ATICH study. EAT biopsies were obtained during open-chest chest surgery. Serum Se was measured by inductively coupled plasma mass spectrometry. Associations between Se and EAT mRNA expression of *Nod-like receptor family pyrin domain-containing protein 3 (NLRP3)* inflammasome components and cytokines, as well as circulating inflammatory markers, were assessed using Spearman’s rho and group comparisons based on median Se levels. Results: Se concentrations were lower in CAD patients than controls (0.9 vs. 1.1 µmol/L, *p* = 0.025). In CAD patients, Se levels correlated with EAT expression of *CASP1* and *IL18*, and with circulating IL-6. Se levels above the median were associated with lower EAT expression of *CASP1* and *NLRP3* and reduced IL-6 levels (*p* < 0.05, all). Our analysis of publicly available RNA seq data demonstrated selenoprotein’s presence in EAT. Conclusion: Lower Se status in CAD was associated with increased systemic and EAT inflammation, suggesting a role for selenoprotein-dependent antioxidant mechanisms in regulating cardiac adipose tissue inflammation.

## 1. Introduction

Cardiovascular disease (CVD), including coronary artery disease (CAD), is a leading cause of death in Europe [1]. Atherosclerosis is the main underlying mechanism of CAD, driven by systemic and local inflammation, endothelial dysfunction, and oxidative stress [2]. Epicardial adipose tissue (EAT) is in direct contact with the myocardium and is implicated in the development and progression of CAD [3,4]. Under physiological conditions, EAT constitutes a protecting depot serving energy to the myocardium. In states of chronic inflammation and/or visceral obesity, often accompanying aging, EAT volume increases and undergoes structural changes with extracellular matrix degradation, the infiltration of immune cells that release pro-inflammatory mediators, and the production of reactive oxygen species (ROS) [5,6,7]. Counteracting the pro-inflammatory milieu is considered therapeutically favorable, e.g., the CANTOS and ASSAIL-MI trials with inhibition of interleukin (IL)-1β and the IL-6 receptor, respectively, show modest cardiovascular risk benefits without a reduction in adverse events in very high-risk patients with residual inflammation [8,9].

Selenium (Se) is an essential trace element [10]. In many European countries, particularly in the Nordic region, low concentrations of Se in the soil contribute to inadequate dietary intake and subsequent risk of Se deficiency [11]. Following dietary intake, Se is absorbed in the distal duodenum and transported to the liver for storage, after which it is distributed to target tissues via the principal Se transporter, selenoprotein P (SELENOP) [12]. Se is incorporated into selenoproteins as the amino acid selenocysteine, and low Se status impairs selenoprotein activity. Twenty-five human genes encode selenoproteins, e.g., glutathione peroxidases (GPXs) and thioredoxin reductases (TXNRDs). Selenoproteins have a wide range of physiological functions, including anti-inflammatory effects such as nuclear factor (NF)-κB inhibition, antioxidant activity such as neutralizing ROS, gene regulation, and the inhibition of endoplasmic reticulum (ER) stress with the removal of misfolded proteins [10,13].

Previous reports show that selenoproteins are highly present in EAT, underpinning their impact in this environment [14,15,16,17,18,19,20].

In the present study, our aim was to explore the role of Se status with regard to its anti-inflammatory effects in CAD, focusing on EAT and circulating proteins. Se status may influence vascular function, atherosclerosis, and CAD severity through the protective effects of selenoproteins against inflammation- and oxidative stress-mediated damage to the cardiovascular system. We focused on innate immunity by assessing the mRNA expression of the Nod-like receptor family pyrin domain-containing protein 3 (NLRP3) inflammasome components in EAT (*NLRP3* and *CASP1*), their related cytokines (IL-1β and IL-18, encoded by *IL1B* and *IL18*, respectively), and *IL6* [21], as well as circulating levels of IL-18, IL-6, and high-sensitivity C-reactive protein (hsCRP), in patients with CAD undergoing coronary artery bypass grafting (CABG).

Our results suggested that Se status in CAD patients was associated with the NLRP3 inflammasome pathway in EAT, not previously reported, as well as systemic inflammatory responses. While omics data including selenoprotein presence in EAT are accessible in the public domain, they have not previously been selectively summarized in a scientific publication. These novel findings support the importance of adequate Se status for the anti-inflammatory and antioxidant functions of selenoproteins in EAT, thereby underscoring their potential relevance in both the prevention and progression of CAD.

## 2. Materials and Methods

### 2.1. The ATICH Study Population

The Adipose Tissue In Coronary Heart (ATICH) trial is an observational, cross-sectional study including *n* = 52 patients with CAD undergoing CABG and *n* = 22 subjects) with valve diseases undergoing valve replacement [22], with the intention to explore inflammatory processes in EAT requiring open-chest surgery for biopsy procedures. Participants were recruited at the Department of Cardiothoracic Surgery, Oslo University Hospital, Ullevaal, Oslo (Norway), from 2016 to 2018. No restrictions were set for inclusion except for use of medications known to interfere with inflammation, e.g., steroid drugs. Before surgery, all patients gave written consent to participate. The study was conducted in accordance with the Declaration of Helsinki, and the protocol was approved by the Regional Ethics Committee of North Norway (#2016/411). The ATICH study is registered at clinicaltrial.gov with the code NCT02760914.

Arterial blood samples were collected before anesthesia. Whole blood was centrifuged at 2500× *g* for 10′ and serum samples were frozen at −80 °C until analysis. During open-chest surgery and prior to initiation of extracorporeal circulation, EAT biopsies were obtained from the area between the right coronary artery and the pulmonary artery. Samples were placed in an RNA stabilizer (Allprotect Tissue Reagent, Cat#76405, Qiagen, Hilden, Germany), before being snap-frozen at −80 °C.

**Figure 1:**
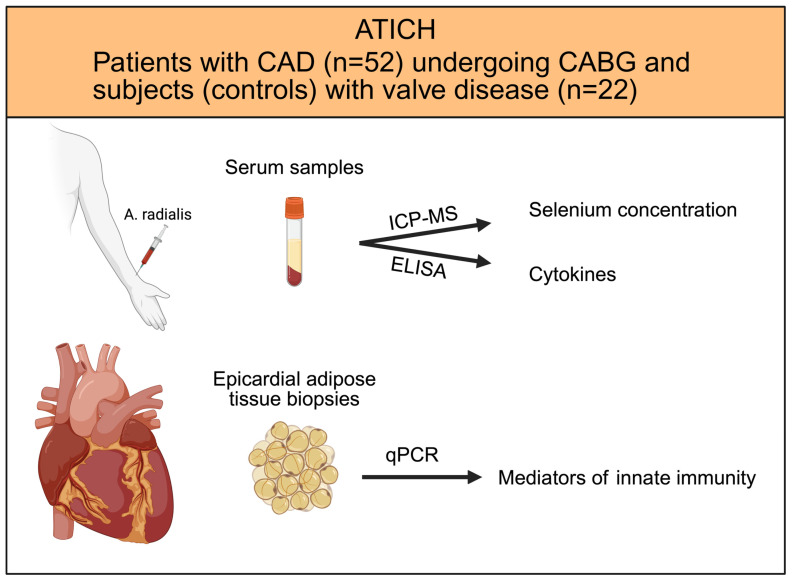
Schematic illustration of the study. The ATICH (Adipose Tissue In Coronary Heart) trial included patients undergoing coronary artery bypass grafting (CABG) (*n* = 52) and subjects with valve diseases undergoing valve replacement (*n* = 22), included as controls. Arterial blood samples were taken from a.radialis, and serum was prepared for Se analysis measured by inductively coupled plasma mass spectrometry (ICP-MS), and serum concentrations of the selected cytokines IL-18, IL-6 and hsCRP. Biopsies from epicardial adipose tissue were obtained between the right coronary artery and the pulmonary artery during open chest surgery from CAD patients and controls. mRNA levels of mediators of innate immunity, the *NLRP3*, *CASP1*, *IL1B*, *IL18*, and *IL6* were measured by qPCR. Created in BioRender. Opdahl, F.L. (2026) https://BioRender.com/9t29sbn.

A schematic illustration of the present study is presented in Figure 1.

### 2.2. Serum Se Measurements

Serum Se was quantified using inductively coupled plasma mass spectrometry (ICP-MS) on a NexlON 2000 B instrument (PerkinElmer, Waltham, MA, USA). Serum samples were diluted 1:20 with water (Milli-RX, Millipore (Millipore Sigma, Burlington, MA, USA), and 0.1% nitric acid, 0.5% butanol (both Merck, Darmstadt, Germany) and 10 ul/L of Rhodium standard (TruQ standard (Perkin Elmer) in 10% HCL) were added to the samples ahead of the mass spectrometry measurements. The precision of the method has an average coefficient of variation (CV) of 7.6%.

### 2.3. ELISA Measurements of Circulating Biomarkers

Serum concentrations of IL-18, IL-6, and hsCRP were determined as part of a previous publication [22]. In brief, commercially available enzyme-linked immunosorbent assays (ELISAs) were used: IL-18 (MBL, Medical and Biological Laboratories Co., Ltd, Nagoya, Japan), Human IL-6 (R&D Systems, Inc., 614 McKinley Place NE, Minneapolis, Minnesota, USA), and C-Reactive Protein HS ELISA (DRG, DRG Instruments GmbH, Marburg, Germany), as previously described [21]. The inter-assay coefficients of variation (CVs) in our laboratory were 1.4%, 2.7%, and 6.1%, respectively.

### 2.4. mRNA Levels in Epicardial Adipose Tissue

Gene expression levels in EAT were determined as part of a previous publication [22]. In brief, total RNA was extracted using the RNeasy Lipid Tissue Mini Kit according to the manufacturer protocol (Qiagen, GmbH, Hilden, Germany). RNA purity and quantity were measured by the NanoDrop 1000 Spectrophotometer (Saveen Werner, Limhamn, Sweden). The mean purity, assessed as the 260 and 280 nm absorbance ratio (260/280), was 1.7, and mean quantity was 28.6 ng/μL. Copy DNA (cDNA) was made from 5 ng/μL RNA with qScript cDNA superMix (Quanta Biosciences, Gaithersburg, MD, USA). The following TaqMan assays were used: *NLRP3* (Hs00918082_m1), *CASP1* (Hs00354836_m1), *IL1B* (Hs01555410_m1), *IL18* (Hs00155517_m1), and *IL6* (Hs00174131_m1), all Applied Biosystems, Foster City, CA, USA). Real-time qPCR was performed on a ViiA7 instrument (Applied Biosystems) using TaqMan^®^ Universal PCR Master Mix (Applied Biosystems, Foster City, CA, USA) (P/N 4324018). B_2_M (Hs99999907_m1) (Applied Biosystems) was used for normalization, and relative quantification (RQ) of the target genes was determined through applying the ΔΔCT method [23].

Publicly available RNA-sequencing datasets from human EAT were identified through a search of the Gene Expression Omnibus (GEO), using the term “epicardial adipose tissue” and applying the filters “homo sapiens” and “expression profiling by high throughput sequencing”. RNA sequencing data were retrieved from GSE108971 [14], GSE125856 [15], GSE192886 [16], GSE222739 [17], GSE179455 [18], GSE135445 [19], and GSE263644 [20]. For the purposes of this analysis, samples were included irrespective of disease status or study group, and all available EAT sequencing data were considered. From the eligible datasets, median transcripts per million (TPM) values for selenoprotein-related genes were extracted and reviewed.

### 2.5. Statistics

SPSS version 31.0 was used for statistics and (SPSS Inc., Chicago, IL, USA) figures were created in GraphPad Prism version 10.6.1 (San Diego, CA, USA) and BioRender (Toronto, ON, Canada). Clinical characteristics are presented as numbers and percentages, mean (SD) or as median values with 25th and 75th percentiles. As most variables had a skewed distribution, non-parametric statistics were used throughout, i.e., Mann–Whitney U test for group comparisons and Spearman Rho for correlation analyses. Linear regression with log-transformed Se levels was used when analyzing the difference in Se concentrations between the CAD and controls, adjusted for age, sex, biochemical markers, and medication, with all variables differently distributed between the two groups. Independent variables added in the model were checked for multicollinearity. Histograms, Q-Q plots and Kolmogorov–Smirnov tests were used to assess the normality of the measured Se concentrations. *p*-values < 0.05 were considered statistically significant.

## 3. Results

### 3.1. Clinical Characteristics

The clinical characteristics of the ATICH study population are summarized in Table 1, i.e., CAD patients and controls with valve diseases. As expected, and described before [22], several comorbidities were more present in the CAD group, with hypertension being the most common, followed by angina, previous myocardial infarction (MI) and any type of diabetes. The CAD group had more men, more use of statins and beta-blockers, and higher levels of glycated hemoglobin A1c (HbA1c) and glomerular filtration rate (GFR) compared with the control group.

### 3.2. Serum Se Concentration in CAD Patients and Controls

**Figure 2:**
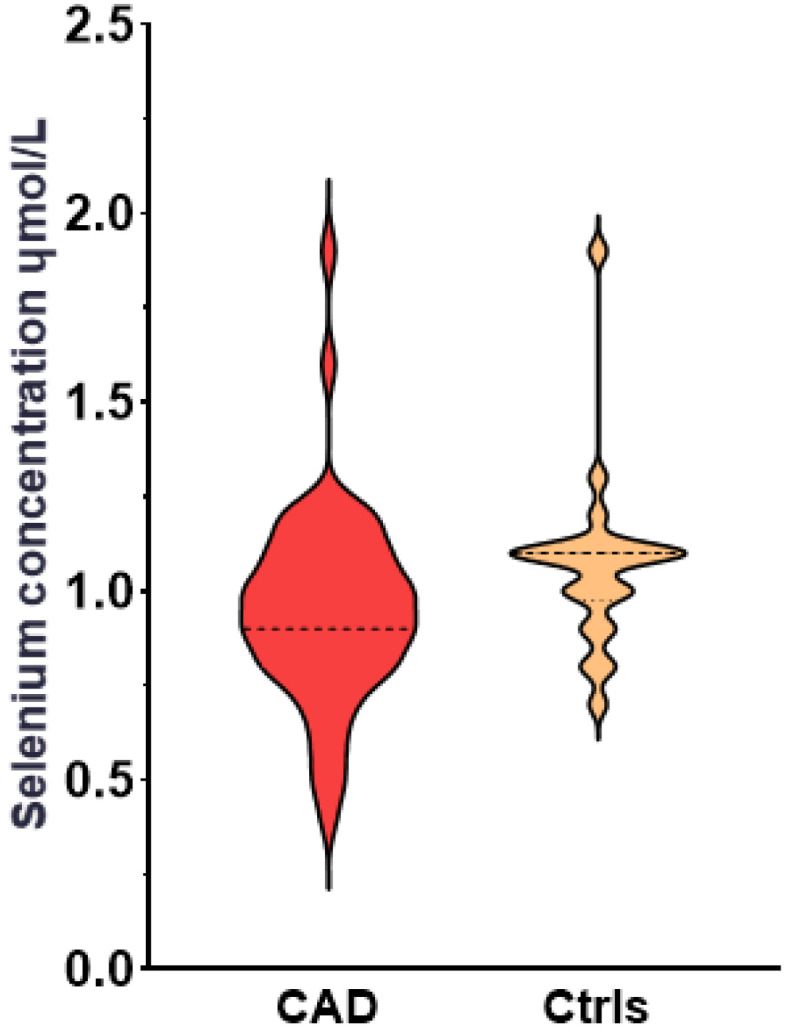
Serum Se concentrations in patients in the ATICH study. Violin plot showing the distribution of Se concentration (µmol/L) in CAD patients and controls. Red plot presents concentrations in the CAD group, median (range): 0.9 (0.4–1.9). Orange plot presents concentrations in the control group, median (range): 1.0 (0.7–1.9). The dashed lines in the figure illustrate the median level.

Se concentrations were successfully analyzed in all seventy-four subjects with medium Se levels (25, 75 percentiles) of 1.0 µmol/L (0.8, 1.1). Se concentrations were lower in the CAD group: 0.90 (0.8, 1.1) compared with controls: 1.1 (0.98, 1.1), *p* = 0.025 (unadjusted). The range (min–max) of serum Se in the CAD and control group were: 0.4–1.9 and 0.7–1.9, respectively (Figure 2).

Of note, when adjusting for age, sex, previous MI, total cholesterol, HbA1c, GFR, and the use of aspirin and beta-blockers in a linear regression model, Se failed to be statistically significantly lower in the CAD group vs. controls, (*p* = 0.141). In the model, previous MI emerged as the strongest and only statistically significant independent contributor to the difference in Se between CAD patients and controls (Table 2). Thus, we speculate that patients with MI may have more severe atherosclerosis, in which Se deficiency could have been a contributing factor.

However, within the CAD group, serum Se concentrations were not associated with comorbidities such as prior MI, diabetes, or hypertension, nor with sex, smoking status or medication use (Table 3).

### 3.3. Association Between Serum Se Concentration and Major Systematic Inflammatory Biomarkers

Correlation analyses of serum Se concentration against mRNA/protein variables (previously measured [22]) were performed only in the CAD group, due to the limited sample size of the control group. For more information, previously measured mRNA levels of the selected genes [22] are shown in Supplementary Table S1. In CAD, serum Se correlated inversely to levels of central inflammation biomarkers IL-6 (*r* = −0.398, *p* = 0.004) and hsCRP (*r* = −0.267, strong tendency, *p* = 0.056), but was not significantly correlated to IL-18 (*r* = −0.069, *p* = 0.63).

**Figure 3:**
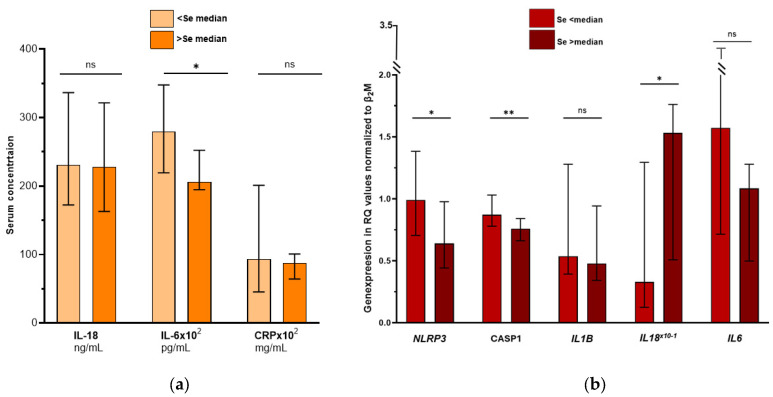
Protein and mRNA variables according to median Se concentration in CAD patients. (**a**) Distribution of cytokines in serum according to median Se levels. Light and dark orange columns indicate Se < and > median levels, respectively. Bars represent 25, 75 percentiles. *p*-values indicate differences in levels of markers according to median Se levels (Mann–Whitney U test) * *p* < 0.05; ns: non-significant; IL: Interleukin; CRP; C-reactive protein. (**b**) Distribution of mRNA levels in EAT according to median Se levels. Light and dark burgundy columns indicate Se < and > median levels, respectively. Bars represent 25, 75 percentiles; *p*-values indicate differences in levels of markers according to median Se levels (Mann–Whitney U test); * *p* < 0.05; ** *p* < 0.01; ns: non-significant; *NLRP3*: Nod-like receptor family pyrin domain-containing protein 3; *CASP1*: Caspase 1; *IL*: Interleukin.

When serum Se concentration was categorized according to median Se level in the total cohort (1.0 µmol/L), the stratified analyses showed lower IL-6 concentration at Se levels > the median (*p* = 0.035), whereas no difference in the levels of CRP and IL-18 was found (*p* > 0.5, both) (Figure 3a).

### 3.4. Associations Between Serum Se Concentration and EAT Expression of the NLRP3-Inflammasome Components and Related Cytokines

In the CAD group, serum Se concentration was inversely correlated to *CASP1* mRNA levels (*r* = −0.335, *p* = 0.015), yet no significant correlation was observed to the expression of the other inflammasome component *NLRP3* (*r* = −0.234, *p* = 0.095) and the downstream cytokine *IL1B* (*r* = −0.002, *p* = 0.98). A positive correlation was observed between serum Se and *IL18* mRNA expression (*r* = 0.300, *p* = 0.032).

Categorizing the expression of the markers according to the median Se level (1.0 µmol/L), *NLRP3* and *CASP1* were lower (*p* = 0.022 and *p* = 0.004, respectively) and *IL18* was more highly expressed (*p* = 0.023) at Se concentrations above vs. below the median level (Figure 3b), yet no association was observed for *IL1B* and *IL6* (*p* ≥ 0.5, both).

### 3.5. Selenoproteins in EAT

Results from the publicly available transcriptome data (RNA sequencing) of EAT, from patients with CAD, heart failure or atrial fibrillation, and diabetes patients, were retrieved from previous publications [14,15,16,17,18,19,20] and are shown in Supplementary Figure S1. Of the 25 genes in the human genome that encode selenoproteins, *GPX1*, *GPX3*, *GPX4*, *SELENOP*, and *SENOW* were observed to be highly expressed in EAT in all seven datasets, with the presence of several others, such as *TXNRD1*, *SELENO-F*, -*M*, and -*N*, also being noted. The selenoproteins *GPX6* and *SELENOV* were not expressed in EAT.

## 4. Discussion

The main findings of this study are that patients with CAD undergoing CABG exhibited lower serum Se concentrations in the unadjusted analysis than valve disease patients, and that Se status was associated with both systemic inflammation and inflammatory gene expression in EAT. Specifically, Se correlated inversely with circulating IL-6 and hsCRP, and CAD patients with Se concentrations above the median had lower IL-6 levels. Moreover, Se status was associated with EAT mRNA expression of key NLRP3 inflammasome components, with lower expression of *NLRP3* and *CASP1* observed at higher Se concentrations.

Patients with valve diseases (including aortic stenosis (*n* = 16), mitral insufficiency (*n* = 5) and mitral stenosis (*n* = 1)), presumably without CAD, were included as controls in the present study. Given their median age of 69 years, the presence of chronic inflammation potentially affecting the coronary arteries cannot be excluded. However, these patients were considered to have a lower inflammatory burden compared with individuals with verified CAD. As the primary aim of the study was to explore inflammation in EAT, the use of biopsies from patients with valve diseases, rather than from healthy subjects, was considered a reasonable and pragmatic approach. Nevertheless, the choice of valve disease patients as controls might have influenced our results and a presumably greater difference in Se levels between CAD and healthy controls might be expected. The adjusted statistical model did not demonstrate a significant difference between the clinical groups, suggesting that comorbidity and disease severity might have influenced the results. This interpretation is supported by the finding that prior MI was the only significant contributor to the difference in Se levels between CAD patients and the control group.

The observed serum Se concentrations (median 0.9 µmol/L in CAD vs. 1.1 µmol/L in controls) are consistent with previous reports demonstrating lower Se status in CAD patients compared with non-CAD patients [24,25]. While some studies have reported comparable Se concentrations between CAD and non-CAD groups, reduced activity of Se-dependent enzymes, such as GPXs, has been observed in CAD patients vs. in healthy controls, and has been associated with disease severity [26,27]. Reviews and recent observational and intervention studies support a cardioprotective role of adequate Se status, associating with reduced cardiovascular morbidity, mortality, and preserved cardiac structure and function [28,29,30,31].

Serum Se concentrations below 0.8 µmol/L are considered low, and CAD subjects in the lower range may therefore have been in a state of Se deficiency with impaired selenoprotein function. Recent reports have indicated that an intake of 120 µg/day is required for the optimal functioning of the most important selenoproteins, such as GPX3 and SELENOP [32,33]. A reduction in the activity of antioxidant selenoproteins, especially GPXs and TXNRDs, may compromise redox homeostasis and promote activation of redox-sensitive inflammatory pathways [34]. Recently, in the EAT of patients with CAD, redox homeostasis was observed disrupted, leading to activation of the NLRP3 inflammasome pathway [35]. This disruption was due to an imbalance of the thioredoxin system, in which the selenoproteins TXNRDs are key components. Several key selenoproteins are highly expressed by immune cells and adipocytes [36,37]. Publicly available transcriptomes indicate the presence of multiple selenoproteins in EAT in patients with CAD, atrial fibrillation, heart failure and diabetes, not previously reported. Especially high expression levels were observed for *GPX1*, *GPX3*, and *GPX4*—all three possessing high antioxidant activity—and the selenium transporter *SELENOP*, and *SELENOW*. The presence of several others was also found, such as *TXNRD1*, *SELENO-F*, -*M* and -*N*, which are mainly involved in protein folding in ER, and gene and antioxidant regulation [14,15,16,17,18,19,20]. *SELENOW* is highly expressed in the heart and skeletal muscle of mice and its expression changed in response to oxidative stress, consistent with redox regulation [38]. GPX4 is essential in preventing lipid peroxidation and for preserving membrane integrity, and appears to be prioritized during Se deficiency in rodents [39]. However, a condition with reduced cytosolic and mitochondrial antioxidant capacity may favor inflammasome activation and local cytokine production, which may directly influence the adjacent myocardium and coronary vasculature due to the lack of a fascial barrier.

Consistent with previous studies [40], we observed lower circulating IL-6 levels in CAD patients with higher Se status. Low Se status has been linked to elevated systemic inflammatory markers, likely reflecting impaired regulation of oxidative stress and inflammatory signaling [40]. Randomized trials and meta-analyses have demonstrated that Se supplementation can reduce circulating IL-6, CRP, and P-selectin [41,42], and experimental and clinical evidence suggests that Se deficiency enhances pro-inflammatory pathways and increases systemic inflammatory responses [43].

We here relate Se status to inflammatory gene expression in EAT. We focused on components of the NLRP3 inflammasome and the downstream cytokines, in which respective proteins, i.e., IL-18 and IL-1β are cleaved into their mature forms by the NLRP3 inflammasome. EAT is metabolically active and enriched in free fatty acids and cholesterol, which can promote NLRP3 inflammasome activation through NF-κB signaling, mitochondrial ROS generation, and lysosomal stress [44,45,46]. Se-dependent antioxidant systems are critical regulators of these processes. Recently, impaired mitochondrial respiratory capacity was observed in EAT in states of CAD progression, which indicates increased ROS production [47]. The lower expression of *NLRP3* and *CASP1* observed at higher Se concentrations may indicate reduced NLRP3 inflammasome activity in EAT, potentially resulting in decreased release of the pro-inflammatory cytokines IL-1β and IL-18 proteins. Suppression of oxidative stress and redox regulation of the NLRP3 inflammasome and related proteins depends especially on the selenoproteins GPXs and TXNRDs, which are found in EAT [48]. Experimental studies also support this molecular mechanism, as Se deficiency has been shown to activate the NLRP3 inflammasome, while Se supplementation suppressed *NLRP3* and *CASP1* expression in vivo [49,50]. The elevated EAT *IL18* mRNA levels observed at intermediate Se concentrations were unexpected and may reflect compensatory or feedback mechanisms in chronic inflammatory states. Although significant differences in EAT were observed according to Se concentrations, the wide confidence intervals of the *IL18* mRNA results raise question of reliability, or biological variance. Notably, *IL18* expression tended to decrease (*p* = 0.07) at Se concentrations above the 75th percentile, suggesting that adequate Se status may be required to limit local inflammation. In this context, a recent study showed that appropriate doses of Se added to standard fodder increased circulating GPX1 and reduced IL-18, IL-6 and TNF-α in serum of hyperthyroid rats [51].

### Limitations

The cross-sectional design precludes causal inference, and Se status was assessed at a single time point. Molecular analyses were restricted to expression analysis of selected targets, without assessments of protein levels or functional activity. Consequently, the findings do not provide evidence of actual protein presence in EAT or of NLRP3 inflammasome activation. The results should therefore be considered hypothesis-generating and warrant further investigation. Additionally, the sample size was modest, and findings may not be generalized beyond an elderly CAD population.

## 5. Conclusions

Patients with CAD exhibited lower serum Se concentrations, which were associated with increased systemic inflammation and enhanced expression of NLRP3 inflammasome components in EAT. Reduced Se availability may impair the selenoprotein-dependent antioxidant defense promoting redox imbalance, NLRP3 inflammasome activation, and pro-inflammatory cytokine production in EAT. Given the close anatomical proximity of EAT to the myocardium and coronary arteries, these inflammatory signals may contribute to adverse cardiac remodeling and atherosclerosis progression. Our data generate novel hypotheses linking Se status to NLRP3 inflammasome regulation in EAT and warrant further investigation.

## Figures and Tables

**Table 1:** Clinical characteristics of the study population.

	CAD Patients (*n* = 52)	Controls (*n* = 22)	*p*-Value
Age (years)	66.5 (62,71.8)	69.0 (63, 71.5)	0.397
Male (%)	40 (76.9%)	11 (50%)	**0.040**
Smoker (previous/current)	31 (59.6%)	10 (45.5%)	0.217
Weight (kg)	85 (70.3, 95.5)	82.5 (77.5, 107.0)	0.445
Height (m)	1.77 (1.68, 1.81)	1.75 (1.67, 1.82)	0.939
Waist (cm)	92 (86, 98)	90 (88, 101)	0.843
Waist to height ratio	0.53 (0.49, 0.56)	0.52 (0.49, 0.55)	0.646
BMI (kg/m^2^)	27.3 (23.8, 30.1)	28.4 (24.6, 31.6)	0.366
SBP (mmHg)	140 (125, 160)	140 (115, 163)	0.825
DBP (mmHg)	80 (70, 87)	79 (70, 86)	0.752
Selenium (µmol/L)	0.90 (0.80, 1.10)	1.1 (0.98, 1.10)	**0.025**
**Cardiovascular status**			
Previous MI (%)	20 (38.5%)	2 (9.1%)	**0.025**
Angina (%)	24 (46.2%)	0 (0%)	**<0.001**
PCI (%)	20 (38.5%)	0 (0%)	**0.002**
Hypertension (%)	28 (53.9%)	9 (40.9%)	0.309
Diabetes type I and II (%)	14 (26.9%)	3 (13.6%)	0.214
Heart failure (%)	3 (5.8%)	1 (4.5%)	1.000
**Medications**			
Aspirin (%)	45 (86.5%)	9 (40.9%)	**<0.001**
Other antiplatelet (%)	14 (26.9%)	0 (0%)	**<0.001**
ACEi/ATII (%)	24 (46.2%)	11 (50%)	1.000
Beta-blockers (%)	32 (61.5%)	6 (27.3%)	**0.015**
Statins (%)	37 (71.2%)	11 (50%)	0.081
Other lipid-lowering agents (%)	10 (19.2%)	1 (4.5%)	0.105
Insulin (%)	6 (11.5%)	0 (0%)	0.232
Anti-diabetic drugs (%)	11 (21.2%)	3 (13.6%)	0.450
Diuretics (%)	7 (13.5%)	5 (22.7%)	0.275
**Laboratory values**			
hsCRP (mg/L)	0.91 (0.49, 1.77)	1.00 (1.00, 2.00)	0.976
IL-6 (pg/mL)	2.56 (2.02, 3.29)	2.45 (1.79, 3.09)	0.390
IL-18 (ng/mL)	230 (171, 335)	223 (182, 279)	0.614
Troponin T (ng/L)	13 (9, 22)	11.5 (9, 25)	0.947
Total Chol (mmol/L)	3.1 (2.7, 3.4)	3.9 (2.8, 4.6)	**0.026**
HDL-Chol (mmol/L)	0.97 (0.75, 1.12)	1.10 (0.87, 1.30)	0.064
LDL-Chol (mmol/L)	1.83 (1.4, 2.2)	2.18 (1.8, 2.9)	**0.020**
Triglycerides (mmol/L)	1.22 (1.0, 1.8)	1.02 (0.9, 1.7)	0.293
Glucose (mmol/L)	5.6 (4.9, 6.6)	5.6 (4.9, 6.3)	0.679
HbA1c (mmol/mol)	39 (36, 51)	36 (33, 38)	**0.011**
GFR (%)	90 (75, 95)	80 (68, 91)	**0.030**
Creatinine (μmol/L)	76 (67, 85)	80 (64.8, 91)	0.434
Uric acid (μmol/L)	313 (271, 362)	317 (258, 396)	0.532

These characteristics and measurements have previously been published as part of the study by Åkra, S. et al. [22]. Levels are median (25, 75 percentiles), or numbers (%). *p*-Values < 0.05 are bolded. BMI: Body Mass Index; SBP: Systolic Blood Pressure; DBP: Diastolic Blood Pressure; MI: Myocardial Infarction; PCI: Percutaneous Coronary Intervention; ACEi: Angiotensin-Converting Enzyme inhibitors; ATII: Angiotensin II receptor antagonist; hsCRP: high-sensitivity C-reactive protein; Total Chol: total cholesterol; HDL-Chol: high-density lipoprotein cholesterol; LDL-Chol: low-density lipoprotein cholesterol; HbA1C: glycosylated hemoglobin A1c; GFR: Glomerular filtration rate.

**Table 2:** Linear multiple regression with log Se concentration as the dependent variable group.

Variable	Std. Coefficient	Std. Error	*t*-Value	*p*-Value
Group	0.239	0.040	1.493	0.141
Age	−0.106	0.005	−0.809	0.422
Sex	−0.141	0.035	−0.982	0.331
Previous MI	−0.304	0.035	−2.140	**0.037**
Total Chol	−0.053	0.020	−0.366	0.716
HbA1c	0.049	0.013	0.037	0.715
GFR	0.125	0.000	0.968	0.337
ASA	−0.059	0.037	−0.410	0.683
Beta-blockers	0.070	0.034	0.471	0.640

The *p*-Value < 0.05 is bolded. MI: myocardial infarction; Total Chol: total cholesterol; HbA1c: glycosylated hemoglobin A1c; GFR: glomerular filtration rate; ASA: aspirin. ANOVA model; *R*^2^: 0.165, VIF: <1.651, all, indicating no multicollinearity.

**Table 3:** Se concentrations in different clinical CAD groups.

Clinical Subgroups	Se Concentration	*p*-Value
Male (40)—Female (12)	1.00 (0.80, 1.10)—0.85 (0.70, 1.08)	0.27
Previous MI Yes (20)—No (32)	0.90 (0.70, 1.10)—1.00 (0.80, 1.10)	0.19
Hypertension Yes (28)—No (24)	1.10 (0.90, 1.10)—0.90 (0.73, 1.08)	0.13
Diabetes type I and II Yes (12)—No (40)	0.95 (0.83, 1.18)—0.90 (0.80, 1.10)	0.42
Smoking status ^a^ Yes (14)—No (38)	0.85 (0.73, 1.08)—1.00 (0.80, 1.10)	0.23
Medication ^b^		
Statins Yes (37)—No (15)	1.00 (0.80, 1.10)—0.90 (0.80, 1.10)	0.68
ASA Yes (45)—No (7)	1.00 (0.80, 1.10)—0.90 (0.60, 1.60)	0.67
Other anti-platelets Yes (14)—No (38)	0.85 (0.80, 0.95)—1.00 (0.80, 1.10)	0.054
Beta-blockers Yes (32)—No (20)	0.90 (0.80, 1.00)—0.95 (0.80, 1.10)	0.51

Number of patients given in the clinical subgroups. Values are median (25, 75 percentiles). *p*-values represent statistical differences between clinical groups (Mann–Whitney U test). MI: myocardial infarction, Diabetes type I and II; ASA: aspirin; ^a^ current smokers vs. no; ^b^ medications known to affects Se absorption or concentration (NIH Office of Dietary Supplements; https://ods.od.nih.gov).

## Data Availability

The raw data supporting the conclusions of this article will be made available by the authors on request.

## Supplementary Materials

The following supporting information can be downloaded at: https://www.mdpi.com/article/10.3390/antiox15060687/s1, Supplementary Table 1; footnote: Added abbreviation for EAT, and CAD Supplementary Figure 1; footnote: Added abbreviations for EAT, and SELENOP/W/M/F/N/H/S/T/K/O/I/V.

## Abbreviations

The following abbreviations are used in this manuscript:

ASSAIL-MI	Anti-Inflammatory Effects of Anakinra in ST-Elevation Myocardial Infarction
ATICH	Adipose tissue in coronary heart disease
CABG	Coronary artery bypass grafting
CAD	Coronary artery disease
CANTOS	Canakinumab Anti-inflammatory Thrombosis Outcomes Study
CASP1	Caspase1
CRP	C-reactive protein
EAT	Epicardial adipose tissue
ELISA	Enzyme-Linked Immunosorbent Assay
GEO	Gene expression omnibus
GFR	Glomerular filtration rate
GPX	Glutathione peroxidase
HbA1c	Glycated hemoglobin A1c
ICP-MS	Inductively coupled plasma mass spectrometry
IL	Interleukin
MI	Myocardial infarction
mRNA	Messenger RNA
NF-κB	Nuclear factor kappa B
NLRP3	Nod-like receptor family pyrin domain-containing protein 3
ROS	Reactive oxygen species
RQ	Relative quantification
SELENOP	Selenoprotein P
TXNRD	Thioredoxin reductase
