# Supplementary material for: Selenium Status Is Associated with Inflammation in Epicardial Adipose Tissue in Elderly Patients with Coronary Artery Disease
Source: Antioxidants (Basel). 2026 May 29;15(6):687. doi: 10.3390/antiox15060687 (PMC13295289; doi:10.3390/antiox15060687)
Supplement: Supplementary file 1 [file antioxidants-15-00687-s001.zip › antioxidants-4254309-supplementary.pdf]

Supplementary material

Supplementary Table 1 EAT gene expression* of selected markers in CAD and controls

Gene	CAD (n=52)	Controls (n=22)	p-value
NLRP3	0.87 (0.53, 1.15)	0.66 (0.36, 1.13)	0.22
CASP1	0.83 (0.73, 0.92)	0.90 (0.80, 0.91)	0.43
IL1B	0.51 (0.33, 1.06)	0.44 (0.27, 1-23)	0.79
IL18	5.70 (1.35, 15.92)	7.40 (1.64, 17.00)	0.49
IL6	1.26 (0.66, 2.23)	0.72 (0.46, 1.63)	0.07

These measurements have previously been published as part of the study by Åkra S et al., [1].
**Arbitrary units. Gene expression was measured with qPCR*
Levels are median (25, 75 percentiles).
p-values refer to difference between CAD and Controls (Mann-Whitney U test).
NKRP3; Nod-like receptor family pyrin domain-containing protein 3, CASP1; Caspase 1, IL; Interleukin

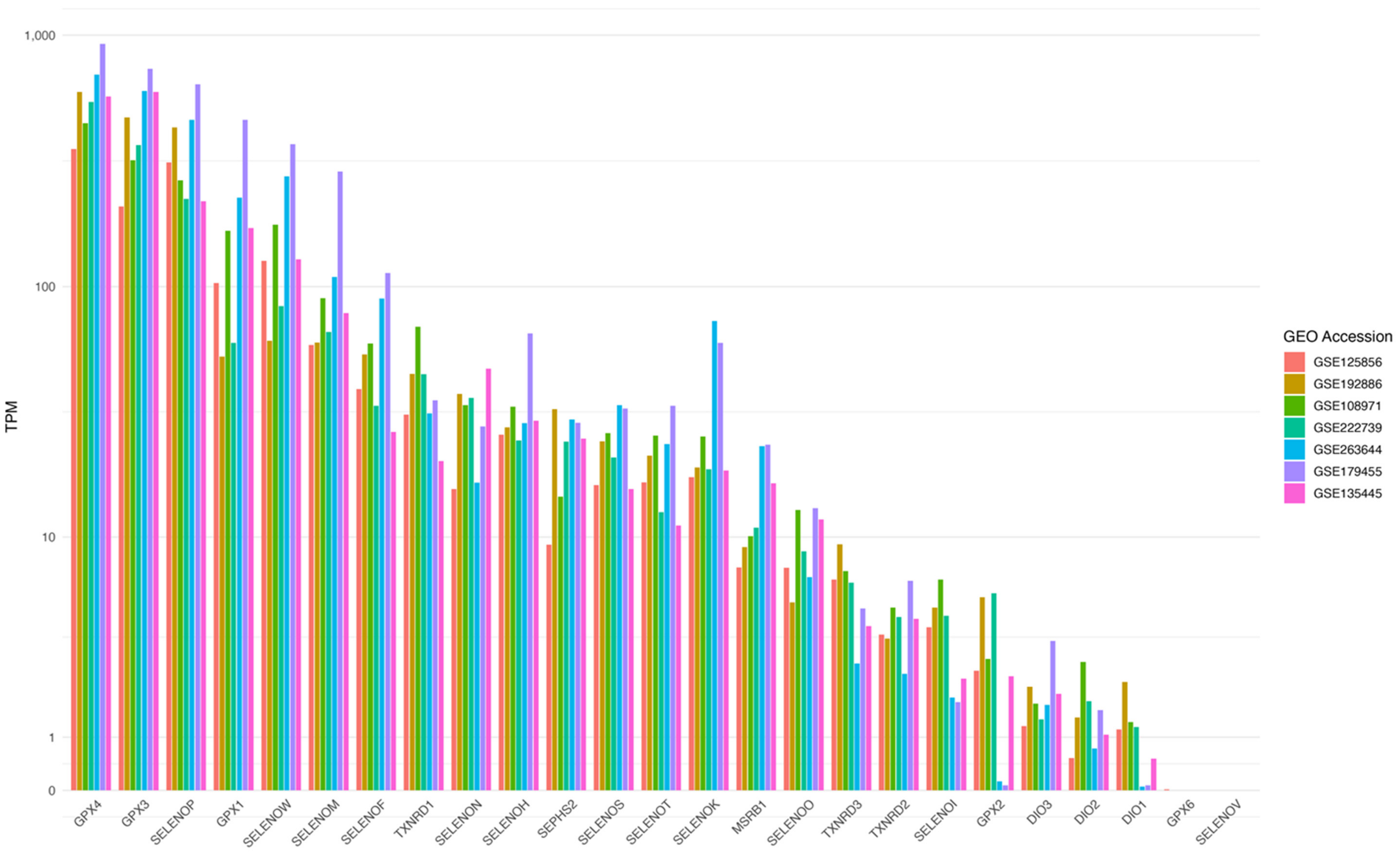

TPM
1,000
100
10
1
0
GPX4
GPX3
SELENOP
GPX1
SELENOW
SELENOM
SELENOF
TXNRD1
SELENON
SELENOH
SEPHS2
SELENOS
SELENOT
SELENOK
MSRB1
SELENOO
TXNRD3
TXNRD2
SELENOI
GPX2
DIO3
DIO2
DIO1
GPX6
SELENOV
GEO Accession
GSE125856
GSE192886
GSE108971
GSE222739
GSE263644
GSE179455
GSE135445

Supplementary Figure 1 Expression of selenoprotein transcripts in EAT

The figure illustrates transcripts per million (TPM) for each selenoprotein transcript in each study:

- GSE125856, Chechi K et al., 2019 [2]
- GSE192886, He S et al., 2023 [16] 3
- GSE108971, Camarena V et al., 2017 [4]
- GSE222739, Peng Y et al., 2024 [5]
- GSE263644, Ryk A et al., 2024 [6]
- GSE179455, Vyas V ret al., 2021 [7]
- GSE135445, Zhao L et al., 2020 [8]

Data are retrieved from the publicly database GEO Omnibus (OMNIBUS).
GPX: glutathione peroxidase, TXNRD: thioredoxin reductase, SEPHS: selenophoshate synthetase, MSRB: Methionine-R-sulfoxide reductase, DIO: Iodothyronine deodinase

References

1. Åkra, S.; Seljeflot, I.; Braathen, B.; Bratseth, V.; Hansen, C.H.; Arnesen, H.; Tønnessen, T.; Solheim, S. The NLRP3 inflammasome activation in subcutaneous, epicardial and pericardial adipose tissue in patients with coronary heart disease undergoing coronary by-pass surgery. *Atherosclerosis Plus* **2022**, *48*, 47–54, doi:https://doi.org/10.1016/j.athplu.2022.03.005.
2. Chechi, K.; Vijay, J.; Voisine, P.; Mathieu, P.; Bossé, Y.; Tchernof, A.; Grundberg, E.; Richard, D. UCP1 expression-associated gene signatures of human epicardial adipose tissue. *JCI Insight* **2019**, *4*, doi:10.1172/jci.insight.123618.
3. He, S.; Zhu, H.; Zhang, J.; Yang, X.; Zhao, L. Genome-wide screening for circRNAs in epicardial adipose tissue of heart failure patients with preserved ejection fraction. *Am J Transl Res* **2023**, *15*, 4610–4619.
4. Camarena, V.; Sant, D.; Mohseni, M.; Salerno, T.; Zaleski, M.L.; Wang, G.; Iacobellis, G. Novel atherogenic pathways from the differential transcriptome analysis of diabetic epicardial adipose tissue. *Nutrition, metabolism, and cardiovascular diseases : NMCD* **2017**, *27*, 739–750, doi:10.1016/j.numecd.2017.05.010.
5. Peng, Y.; Su, P.; Zhao, L. Long noncoding RNA and messenger RNA profiling in epicardial adipose tissue of patients with new-onset postoperative atrial fibrillation after coronary artery bypass grafting. *Eur J Med Res* **2024**, *29*, 134, doi:10.1186/s40001-024-01721-x.
6. Ryk, A.; Marcinkiewicz, A.; Chrzanowski, J.; Michalak, A.M.; Dróżdz, I.; Burzyński, J.; Krejca, M.; Fendler, W. Cholesin receptor signalling is active in cardiovascular system-associated adipose tissue and correlates with SGLT2i treatment in patients with diabetes. *Cardiovasc Diabetol* **2024**, *23*, 211, doi:10.1186/s12933-024-02322-y.
7. Vyas, V.; Blythe, H.; Wood, E.G.; Sandhar, B.; Sarker, S.J.; Balmforth, D.; Ambekar, S.G.; Yap, J.; Edmondson, S.J.; Di Salvo, C.; et al. Obesity and diabetes are major risk factors for epicardial adipose tissue inflammation. *JCI Insight* **2021**, *6*, doi:10.1172/jci.insight.145495.
8. Zhao, L.; Ma, Z.; Guo, Z.; Zheng, M.; Li, K.; Yang, X. Analysis of long non-coding RNA and mRNA profiles in epicardial adipose tissue of patients with atrial fibrillation. *Biomed Pharmacother* **2020**, *121*, 109634, doi:10.1016/j.biopha.2019.109634.
